# Curing or causing? HIV/AIDS in health care system of Punjab, Pakistan

**DOI:** 10.1371/journal.pone.0254476

**Published:** 2021-07-09

**Authors:** Muhammad Ahmed Abdullah, Babar Tasneem Shaikh, Haider Ghazanfar

**Affiliations:** 1 Islamabad Medical & Dental College, Islamabad, Pakistan; 2 Health Services Academy, Islamabad, Pakistan; 3 Shifa International Hospital, Islamabad, Pakistan; Marie Stopes International, PAKISTAN

## Abstract

**Background:**

Pakistan’s National AIDS Control Program has registered 44,000 HIV/AIDS patients to date, but the actual number of cases have been estimated to be as high as 150,000–170,000. The health care system has a very important role to play in this equation and must be reformed to improve the health care services in Pakistan, with regards to HIV/AIDS.

**Methods:**

It was a qualitative research employing a phenomenological approach. The principal researcher visited nine public and private health care facilities and conducted 19 key informant interviews with people working for providing preventive and curative services, in addition to the observations made on the site.

**Results:**

Pakistan’s health system has a limited capacity to address the HIV spread in the country, with its current resources. There is an obvious scarcity of resources at the preventive, diagnostic and curative level. However, menace can be curtailed through measures taken at the service delivery level by checking the unsafe needles practices, unclean surgical procedures and an unregulated and untrained private health workforce which are dangerous potentials routes of transmission of the virus to the general population. Healthcare establishments carry the chances of nosocomial infections including HIV/AIDS. Poverty, illiteracy and stigma associated with the disease is compounding the overall situation.

**Conclusion:**

Improved accessibility to service delivery with a greater focus on prevention would be imperative to address the threat of HIV/AIDS in Pakistan. A health systems approach would help in identifying gaps at both strategic and operational levels, and concurrently find and implement solutions.

## Background

Brushing problems and stressful thoughts under the carpet is a legitimate response on an individual basis, according to Diagnostic and Statistical Manual of Mental Disorders (DSM 5) [1]. However, a similar response on a larger scale disrupts the efforts that might discover solutions [2]. It is still commonly observed that HIV/AIDS is not discussed very openly in a comfortable environment, even among the medical and scientific fraternity, which affects logical planning and strategy to counter HIV/AIDS in Pakistan [3]. Hence the prevalence of HIV/AIDS in the country, if not on the alarming rise, has been persistently escalating over the past three decades [4]. The national surveillance data suggests that the three major sources of spread of HIV in the country are commercial sex, injectable drugs and unsafe blood transfusions [5–7], amidst a weak national screening program for HIV. The sense of innate protection against the virus based on religious dogmas and socio-cultural values is thus hard to comprehend [8].

Pakistan’s HIV epidemic has been declared to be in the concentrated phase, which means less than 1% cases in the general population. However, the major high risk groups (HRGs) exhibiting prevalence higher than 5% which include commercial sex workers (male, female, transgender), injectable drug users, and the repatriated migrant workers. The estimates of people living with HIV/AIDS (PLHIV) are much higher as compared to the actual number of registered cases, because of inadequate flow of information and sparse diagnostic facilities. Stigma attached with the disease further aggravates the situation [9]. In a country with a low literacy rate, where many health perceptions are governed by myths and fallacies, and with a grossly under-utilized public health system; diseases such as HIV attain much greater magnitude based on the financial, social and political hurdles [10].

Pakistan’s National AIDS Control Program (NACP) has registered approximately 44,000 cases till now, but the actual number has been estimated to be as high as 150,000–170,000 [11]. The health care delivery system has a very important role to play in this equation. The NACP provides preventive, diagnostic and therapeutic services through the hospital based infrastructure in the country. However, the departments assigned with this task, function in a state of isolation; most often due to lack of motivation and planning, and sometimes to avoid stigmatization of their clientele [12]. The NACP, in collaboration with its provincial counterparts, has set up 19 HIV treatment centers all of which are based in hospitals. These centers provide diagnostic, counseling and treatment services to individuals who volunteer or are referred there. In addition, seven centers for the Prevention of Parent to Child Transmission of HIV have also been set up in the country. However, a major disconnect is present between the mainstream health care delivery system and the specialized units for HIV/AIDS. The issue of hospital/healthcare acquired infections adds further aggravates the problem. Medical and surgical procedures lack robust sterilization techniques; and the dental practices require deep rooted attention in this regard. The shabbily managed healthcare waste disposal system is another important block in the puzzle, along with unsafe injection prescriptions and a huge workforce of unregistered medical practitioners [13]. In various surveys conducted in different parts of the country, major gaps have been observed in the knowledge and practices of registered medical practitioners as well as among the medical students [14,15]. The larger study looks into the prospects of improving the health care services in Pakistan, with regards to HIV/AIDS, through the lens of the WHO’s six Health Systems Building Blocks Framework, however, this paper focuses more on the service delivery and human resource. With this state of affairs in the backdrop, this study endeavored to understand the dynamics of the present healthcare delivery system vis-à-vis HIV/AIDS in Pakistan and to identify possible sources of hospital acquired infections. It is envisaged that addressing these two broader objectives will answer our research question: “Does the health system of Pakistan have the capacity to limit the current state of the HIV/AIDS epidemic, and what changes are needed to improve the current system?”.

## Methods

This paper is based on the qualitative part of the larger mixed-methods study. The principal investigator visited public and private health care delivery facilities and conducted key informant interviews with health personnel providing HIV related services. To answer many of the above mentioned questions, if not all, the present qualitative research study was designed, employing a phenomenological approach with two main purposes: i) to understand the personal perceptions, subjective experiences and to generate an opinion about the current healthcare delivery practices for preventing and managing HIV/AIDS in hospital settings of Pakistan, and ii) to identify possible sources of hospital acquired infections. Steps included in the phenomenology approach were bracketing the interpretation of the question by the respondent; delineating the response; clustering of similar responses, extracting and generating generic as well as unique themes. All interview notes were transcribed within 48 hours by the principal researcher (a doctoral candidate and an experienced public health researcher). The information hence gleaned was analyzed manually and a thematic content analysis was done to generate relevant themes.

Moreover, there were certain observations made on the site by the researcher/principal investigator. The main sites visited during this research included Benazir Bhutto Hospital, Naz Male Health Association, Behtar Kal Program (Rawalpindi), Aziz Bhatti Shaheed District Headquarters hospital (Gujrat), Civil Hospital, New Lights AIDS Control Society, Jallalpur Jattan Blood donors private blood bank (District Gujrat), Dera Ghazi Khan Medical College Hospital and District Headquarters Hospital (District Lodhran). The above sites were selected because of the maximum concentration of HIV cases reported and registered here. The observations of the principal investigator have also been presented to discuss the gaps identified in the present health care delivery system with regards to HIV/AIDS in Pakistan; this has been done through a reflexivity-based approach. The study got approval from the Institutional Review Board of the Health Services Academy, Islamabad. A letter was sent to all heads of the hospitals to be visited informing about the objectives of the study. Each interview was conducted by the principal investigator in the hospital settings after obtaining permission from the respective hospital administration, and written informed consent from the study participants. The names, designations and institutional affiliations have not been mentioned to respect respondents’ confidentiality. The sample size was determined with the point of saturation reached and when no new information or perspective was added.

A semi structured questionnaire was used for conducting the interviews. The interviews took around 30–45 minutes each and were audio recorded in most instances. The recordings were then transcribed into text within 48 hours of the interview. The interviews were based on two main premises under consideration. Firstly, to assess the current capacity of the health system to control the spread of HIV in Pakistan, and secondly to identify the healthcare malpractices and other gaps in service delivery that could potentially extrapolate the transmission of HIV among the general public.

## Results

Researchers interviewed 19 public and private health professionals (men: 12; women: 7) providing preventive and curative services in the HIV/AIDS program of Pakistan. They were mostly medical officers, senior registrars and blood bank managers in case of government hospitals and project managers, field managers, Sexually Transmitted Infections(STI) specialists and project directors in case of NGOs/private institutions. They were all in the age bracket of 30–55 years. Private sector providers were added because there is a pre-dominant health care seeking in private sector in Pakistan. The response rate was 100% and no refusals were faced.

The delivery of curative and preventive services for HIV/AIDS is managed through various levels of the health system. The public and private sectors both play their part in this regard. Table 1 elaborates the strengths and weaknesses identified vis-à-vis service delivery.

**Table 1:** Service delivery analysis based on key informant interviews.

Types of Services	Service providers	Strengths	Weaknesses
**Preventive**	NGOs Public sector Voluntary Counseling &Testing centers	• Targets high risk groups • Health education • Use peer education as a more acceptable approach • Keeping a broader reproductive health paradigm and not targeting HIV/AIDS alone • Condom and autodestruct syringe distribution for harm reduction	• Scarce resources (financial as well as human) • Inadequate use of print, electronic and social media • Taking political advantage by showing support to the program • Lack of attention to general population • Difficult to reach HRGs • Social barriers such as illiteracy, poverty, ignorance on causes of disease, stigmatization etc. • Denial of issues such as burden of disease, prevalence, causes of spread, homosexuality etc. • Social exclusion of HRGs • Poor flow of information
**Diagnostic**	NGOs Public sector Private sector	• Respondent driven sampling • Gate keepers • Incentive based approach to increase the case detection	• Limited resources (financial and human) • No follow up mechanism • Lack of proper reporting (Health Management Information System) • No structured mechanism for contact tracing • No mechanism for regular screening of HRGs • No regular screening for people entering Pakistan • Fear of stigma and discrimination if found getting tested
**Curative**	Public sector	• Strong and wide spread healthcare infrastructure to cover the HRGs	• Limited resources (financial and human) • Limited workforce capacity and lack of motivation • Poor flow of finances to the operational level • Health care waste management issues • Greater chances of hospital acquired infections • Extremely limited availability of treatment options • Stigma attached with seeking care

Some additional subthemes identified under the Service Delivery have been discussed below:

- **Preventive Services:** It was learned that preventive services generally lack robustness, and are liable to various alterations due to unstable policies and donor dependence. The periodic interruptions in the funding and implementation agendas were found to be issues requiring close attention.
  *“We get involved in some projects on HIV prevention whenever a donor funded activity comes in; but then it phases out and activities also are not sustained”*. *[Field Manager, NGO]*
- **Diagnostic Services:** Since Pakistan has a concentrated HIV epidemic and the disease is in low prevalence in the general population, therefore, regular mass screening campaigns are not conducted, and diagnostic investigations are carried out based on suspicion or referral. This being the case, the health system can only cater to the tip of the iceberg that seeks care for illnesses, ignoring the hidden base. The private sector provides diagnostic services, however physical, financial and social accessibility of the suspected cases were identified as the major impediments in early diagnosis and prompt treatment.
  *“There is a common perception that diagnostic services are expensive, therefore, common man avoids to the extent possible and also because of fear of being isolated by the community”*. *[STI Specialist, NGO]*
- **Treatment Facilities:** Antiretroviral drugs are available only at the public sector treatment centers around the country. This process improves the chances of patient registration and drug compliance due to regular follow up visits, and also reduces the threat of drug resistance. Concurrently, it limits the accessibility of individuals from far flung areas. The issue of stigmatization of people attending HIV clinics, hampers their early diagnosis.
  *“Luckily, AIDS treatment is available in government hospitals and is free. We keep the record of the patient, follow up and keep an eye on the compliance so as to avoid drug resistance”*. *[Registrar, Public sector]*
- **Referral Services:** Referral of patients is generally non-functional in Pakistan’s health care system, especially due to the lack of a structured system and proper feedback mechanism. These services are also misused in certain instances, where unnecessary referrals are made. The lack of a team approach and absence of inter-sectoral collaboration were identified as serious issues in this context.
  *“One hospital is not linked with other hospital; there are seldom any connectivity, so it always uncertain whether a patient referred will be received at the other end or not*.*” [Assistant Professor/Researcher, Public sector]*
- **Hospital Acquired Infections:** It is a pertinent issue not only with regard to HIV/AIDS but also for other blood borne infections such as Hepatitis B and C. Dental treatments, gynecological and obstetric procedures, surgical interventions, renal dialysis and blood transfusions are responsible for the spread. The conundrum of poor sterilization practices and the insensitive attitude of healthcare staff towards the standard operating protocols are major problems. One respondent serving at a large public sector tertiary care hospital narrated the following incident:
  *“We received a patient in urgent need of dialysis in our unit. Due to the shortage of time we sent his screening tests (HBV, HCV and HIV), and initiated required treatment. Once the patient was stable, we referred him to a larger tertiary care setup. We initiated the dialysis of other patients once he had left. Without sterilizing the machine or checking the follow up result; which was HIV positive. By that time many others had received dialysis from the same machine*.*” [Medical Officer, Public sector]*
  Another respondent told us that:
  *“We have separate dialysis units for infected and non-infected patients, but this means that someone infected with hepatitis B has the risk of getting Hepatitis C or HIV in addition to what he/she already has*.*” [Medical Officer, Public Sector]*
  Regarding sterilization practices, another statement recorded says
  *“It is better to come early in the morning for a dental procedure, that is when most equipment is sterilized; the chances of reuse increase as the day passes by.” [Deputy Medical Superintendent*, *Public sector]*
- **Healthcare Waste Management:** This aspect still needs a lot of work in Pakistan. The infectious waste generated from healthcare activities is often, if not always, mixed with other solid waste and is dumped at various dumping sites. It is a common sight that young street children are scavenging for various recyclable materials at the large dumping sites in Islamabad. We discovered syringes, sharp needles and other infectious material which could potentially cause injury to these scavengers. While talking to the administrator of a government hospital, we recorded:
  *“We do not have exclusive budget line for disposal of these infectious waste products; so these are collected by the sanitary workers along with the other hospital wastes. They dispose it off at their will at a nearby place.” (Medical Superintendent*, *Public sector)*
- **Contact tracing and Partner Notification**: These are essential components of HIV/AIDS service delivery. However, both of these imperative tasks are not practiced in a structured manner, owing to the lack of outreach facilities, sometimes due to social and ethical issues and at times due to poor training and lack of motivation of staff. One respondent from a large public sector hospital near a major HIV pocket shared:
  *“We do not trace the contacts of the patients, we ask about the mode of HIV transmission from all patients, but we can’t force anyone to tell the truth. We do not have enough resources for reaching out to the people in the communities.” [Assistant Professor*, *Public Sector]*
- **The Unregulated Private Sector:** The public health care delivery system in Pakistan has always faced resource constraints. The private sector hence plugged in the gaps of service delivery and hence a large majority of people seek care at private health facilities. Furthermore, owing to rampant illiteracy, many people visit unregistered and unqualified medical practitioners, especially in rural and peri-urban areas. These sham healers include a vast array of roadside dental practitioners, and complementary and alternative medical practitioners employing poor needle safety practices; and the traditional birth attendants using unclean delivery kits during childbirth procedures. They are one of the major sources of the spread of blood-borne infections.
  *“HIV/AIDS and other infections spread largely because of private unlicensed non-qualified quacks; unless government brings a stringent law for controlling them*, *the problem will exist.” [Senior Registrar, Public sector]*
- **Reaching the High Risk Groups:** The public sector lacks the capacity to reach out to the HIV high risk groups in Pakistan. Therefore, most of the outreach work related to HIV prevention and control is being carried out by the NGOs. These organizations provide health education, free condoms and new syringes, and diagnostic and referral services. The public sector has taken up the burden of providing the curative services exclusively.
  *“Most of the outreach work is done the NGOs in Pakistan. Whenever we have a project, we provide services to our catchment communities.” [Field Manager*, *NGO]*

## Discussion

There is a need to delve into deeper programmatic and financial analysis to ascertain whether the HIV/AIDS program in Pakistan faces a shortage of resources, and if it is true; of course policymakers and program managers must look into the issue, either by injecting more money or by making judicious use of present allocated money. This may also resolve the issue of capacity building needs of the workforce involved in the HIV/AIDS program. Donor dependence of the AIDS program has largely affected the preventive part and hence the periodic surges of HIV/AIDS have been observed in the country and new hot spots emerged. The fact that the health system, both public and private, has gaps in service delivery, raises the need to gear up the efforts, pool and maximize the resources to cope with the threat. This calls for a large scale mass screening of the population living n and around the identified hot spots. Largely unregulated medical practices, casual attitude towards sterilization, and in the absence of mandatory continuing education for the health workforce, the HIV/AIDS threat remains imminent. Service delivery for HIV/AIDS needs to be expanded beyond the larger urban centers and must cover all the concentrated pockets and vicinities of the population, as shown in the national data. If resources do not permit to do so, then a systematic and functional referral system should be in place to facilitate the suspected cases to reach the nearest health facility for screening. Major health facilities treating HIV patients need radical measures in place for infection prevention [16]. Health Care Commissions established recently in all provinces in Pakistan have been mandated to check on the service delivery standards of all public and private healthcare establishments and this is a good omen for curbing the menace of hospital acquired infections. They ought to focus on the issues of hospital waste management and associated potential infections, particularly in the public sector, and ensure the implementation of standard operating procedures.

Personal Resilience and Enrichment Programs promote interventions that are meant to improve the psychological well-being of various HIV high risk and marginalized groups, and facilitate them to adopt safe practices, to decrease HIV transmission [17]. Given the fact that harm reduction plays a pivotal role in the context of HIV/AIDS, high quality condoms in appealing packaging backed up by effective yet socio-culturally contextualized communication and advertising can reduce HIV transmission in the population. It is important that availability of the condoms should not be limited to pharmacies only and all possible retail outlets should be displaying the products for the ease of accessibility [18]. Community mobilization and participation have also been declared as successful interventions for destigmatizing the diseases and for curbing the menace of many infectious diseases, not just HIV [19]. A systemic review covering different community programs concluded that community empowerment programs were unable to progress from the initial stages due to disunity among community members, variation in the level of commitment of programs and resource limitations [20].

Due to the scarcity of time and budget for the research, the study faced a big limitation of not being able to involve the provincial managers and health care providers in the HIV/AIDS program as well as the stakeholders from other sectors.

## Conclusion

No doubt, the current state of affairs regarding HIV/AIDS is worrisome, as most health care facilities focus only on diagnostic and treatment levels, while prevention remains a distant priority. Improved accessibility to service delivery; enhanced, timely and dependable flow of pertinent information; a self-sustainable funding model based on financial and social risk protection needs to be enforced to tackle the impending threat of a generalized HIV epidemic in Pakistan. The use of a health systems approach is imperative in this regard, which would simultaneously work to identify gaps at both strategic and operational levels, and concurrently find and implement solutions.
